# Treg cells mediate recovery from EAE by controlling effector T cell proliferation and motility in the CNS

**DOI:** 10.1186/s40478-014-0163-1

**Published:** 2014-12-05

**Authors:** Michail Koutrolos, Kerstin Berer, Naoto Kawakami, Hartmut Wekerle, Gurumoorthy Krishnamoorthy

**Affiliations:** Department of Neuroimmunology, Max Planck Institute of Neurobiology, Am Klopferspitz 18, 82152 Martinsried, Germany; Institute of Clinical Neuroimmunology, Ludwig-Maximilians-University, Marchioninistr. 15, 81377 Munich, Germany

**Keywords:** Regulatory T cells, 2-photon imaging, Experimental autoimmune encephalomyelitis

## Abstract

Regulatory T cells are crucial in controlling various functions of effector T cells during experimental autoimmune encephalomyelitis. While regulatory T cells are reported to exert their immunomodulatory effects in the peripheral immune organs, their role within the central nervous system (CNS) during experimental autoimmune encephalomyelitis is unclear. Here, by combining a selectively timed regulatory T cells depletion with 2-photon microscopy, we report that regulatory T cells exercise their dynamic control over effector T cells in the CNS. Acute depletion of regulatory T cells exacerbated experimental autoimmune encephalomyelitis severity which was accompanied by increased pro-inflammatory cytokine production and proliferation of effector T cells. Intravital microscopy revealed that, in the absence of regulatory T cells, the velocity of effector T cells was decreased with simultaneous increase in the proportion of stationary phase cells in the CNS. Based on these data, we conclude that regulatory T cells mediate recovery from experimental autoimmune encephalomyelitis by controlling cytokine production, proliferation and motility of effector T cells in the CNS.

**Electronic supplementary material:**

The online version of this article (doi:10.1186/s40478-014-0163-1) contains supplementary material, which is available to authorized users.

## Introduction

CD4^+^Foxp3^+^ regulatory T cells (T_reg_) have a well-characterized role in promoting peripheral immunological tolerance throughout life by suppressing deleterious inflammatory responses [1]. Lack of T_reg_ due to mutations in the FOXP3 gene in humans results in aggressive multi-organ autoimmunity called IPEX (immunodysregulation, polyendocrinopathy, enteropathy, X-linked) syndrome [2]. Similarly, *scurfy* mice, which harbor mutations in the *Foxp3* gene, or *Foxp3*-gene deficient mice suffer from a massive lymphoproliferative syndrome [3,4]. Targeted depletion of T_reg_ also resulted in severe multi-organ autoimmunity [5,6]. Intriguingly, however, no spontaneous central nervous system (CNS) inflammation was observed in *Foxp3* mutant mice or after targeted depletion of *Foxp3*+T_reg_ cells in wild type mice [7].

T_reg_ have been demonstrated to be capable of controlling CNS autoimmunity in several Experimental Autoimmune Encephalomyelitis (EAE) models. The frequencies of T_reg_ population within the CNS were elevated during the recovery phase of actively induced EAE [8-10]. Moreover, several studies described that the transfer of CD25^+^ T_reg_ ameliorated EAE symptoms [9,11-13]. In addition, non-specific ablation of natural T_reg_ by anti-CD25 antibodies has been reported to exacerbate EAE [9,14-16]. Furthermore, T_reg_ have been shown to prevent spontaneous EAE development [17,18] or delay spontaneous EAE onset [19].

Where and how do T_reg_ exert their control over myelin-specific T cells? In principle, T_reg_ could suppress effector T cells (T_eff_) in the periphery or within the target organ, CNS. One report demonstrated that T_reg_ accumulate in the CNS at the peak of EAE but were unable to suppress CNS-derived T_eff_*in vitro* [8]_._ In contrast, T_reg_ isolated from the recovery phase of the disease were still capable of suppressing T_eff_ [10]. Furthermore, another study reported that, in the absence of T_reg_, there is an enhanced migration of T_eff_ from the periphery [19]. T_reg_ are known to limit the inflammatory reactions using several mechanisms that include soluble mediators, cell-to-cell contact with T_eff_ or inhibiting antigen presenting cells (APCs) [1]. T_reg_ influence EAE by affecting the priming and polarization of T_eff_ [11,20]_._ Among soluble cytokines produced by T_reg_, IL-10 is important in containing T_eff_ proliferation *in vitro* [16]. T_reg_ can also set a threshold for activation of autoreactive T_eff_ by inhibiting their contacts with antigen-loaded dendritic cells (DCs) in the lymph nodes [21-23]. Furthermore, T_reg_ have been shown to contact and inhibit DCs *in vitro* via CTLA-4 [24]. However, the mode of action of T_reg_ during CNS autoimmunity, in particular within the target organ, still remains unclear.

To address those principal outstanding issues, in the present study, we combine targeted and acute depletion of T_reg_ with intravital two-photon microscopy to investigate the functional role of T_reg_ in the CNS during EAE. We found that T_reg_ limit autoimmune inflammation by controlling the T_eff_ proliferation and motility within the CNS.

## Material and methods

### Animals

DEREG [6] and T-Red [25] mice with the C57BL/6 genetic background were used. All mice were bred in the animal facility of the Max Planck Institute of Neurobiology and all experiments were conducted according to the guidelines of the committee on animals of the Max Planck Institute of Neurobiology and were approved by the Regierung von Oberbayern.

### EAE induction and diphtheria toxin treatment

EAE was induced by injecting the mice subcutaneously into the flanks with 200 μl of emulsion containing 200 μg MOG_35–55_ peptide (MEVGWYRSPFSRVVHLYRNGK) and 500 μg *M. tuberculosis* strain H37 Ra (Difco) in incomplete Freund Adjuvant oil (Difco). In addition, the mice received 400 ng pertussis toxin (List Biological Laboratories) intraperitoneally (i.p.) on days 0 and 2 after immunization. Clinical signs of EAE were assessed daily according to the standard 5 point scale [26]. For depletion of T_reg_ in DEREG mice, diphtheria toxin (Sigma-Aldrich) was injected both i.p. and i.v. (200 ng respectively) on day 4 post EAE onset.

### Cell isolation and flow cytometry

Cells from lymph nodes and spinal cord were isolated as described before [26]. For detection of cell surface markers, cells were stained in FACS buffer (PBS containing 1% BSA and 0.1% NaN_3_) with the following fluorochrome labeled monoclonal antibodies: anti-CD45 (30-F11), anti-CD4 (RM4-5), anti-CD25 (PC61) and anti-CD44 (IM7). For intracellular cytokine staining, cells were incubated for 16 hours with anti-CD3 (0.5 μg/ml). Next, cells were fixed and permeabilized by incubation with Foxp3 Fixation/Permeabilization Buffer (eBioscience) and stained in Permeabilization Buffer (eBioscience) with the following fluorochrome labeled monoclonal antibodies: anti-Foxp3 (FJK-16s), anti-IL-17 (eBio17B7) and anti-IFNγ (XMG1.2). All antibodies were purchased from BD Pharmigen or eBioscience. For cell number quantification, 10^4^ FACSuite FC Beads (BD) were added per sample prior to acquisition. Samples were acquired on FACS Verse (BD). FACS data were analyzed using FlowJo 7.6.5 software (TreeStar).

### EdU proliferation assay

For *in vivo* proliferation experiments, 400 μg EdU (Life Technologies) were injected i.p. to mice ~16 hours before their sacrification. The Click-iT® EdU Alexa Fluor® 647 Flow Cytometry Assay Kit (Life Technologies) was used for staining for flow cytometry according to manufacturer’s instructions.

### Immunofluorescence

The organ sections were prepared as described previously [27]. The following monoclonal antibodies were used for staining: biotin-anti-CD4 (RM4-5; BD), Alexa Fluor 647-anti-CD11b (M1/70; Biolegend), Alexa Fluor 488-anti-Foxp3 (FJK-16 s; eBioscience), and Alexa Fluor 568-streptavidin (Invitrogen). Images were acquired on a SP5 confocal microscope (Leica), using 20x air-immersion (N.A. 0.70) or 63x oil-immersion (N.A. 1.4) objective. Images were processed using Image J (NIH) and Photoshop CS5 software (Adobe Systems).

### *In vivo* IL-2 blocking

MOG_35–55_/CFA-immunized DEREG B6 mice were treated with DTx, as described above. Purified anti-IL2 (JES6-1A12) monoclonal antibody or isotype control antibody (J1.2) was injected i.v. on day 4 (400 μg) and day 6 (200 μg) post EAE onset.

### Intravital imaging

The technical setup of the 2-photon microscopy was as described before [28]. The pulsed laser was tuned to 880 nm and routed through a 25× water immersion objective (N.A. 0.95, Leica). Typically, a field of 360 × 360 μm was scanned, and 40–80 μm *z*-stacks were acquired using a 3–6 μm *z*-step. The acquisition rate was set to 25.219 s intervals, with images line-averaged twice. The fluorescence signals were detected using non-descanned photomultiplier tube (PMT) detectors (Hamamatsu) equipped with 525/50 nm (for detection of Alexa Fluor 488) and 630/69 nm (for detection of dsRedII) band-pass filters (Semrock). Mice were anesthetized and imaging in the spinal cord was performed as described previously [28]. For labeling of perivascular meningeal APC, we performed local instillation of Alexa Fluor 488–conjugated dextran (10 ng/μl, 10 kDa; Life Technologies) 20 min prior to imaging, as described before [29]. Image analysis was performed as described previously [29].

### Statistical analysis

Statistical evaluations were performed as indicated in figure legends using GraphPad Prism software.

## Results and discussion

**Figure 1:**
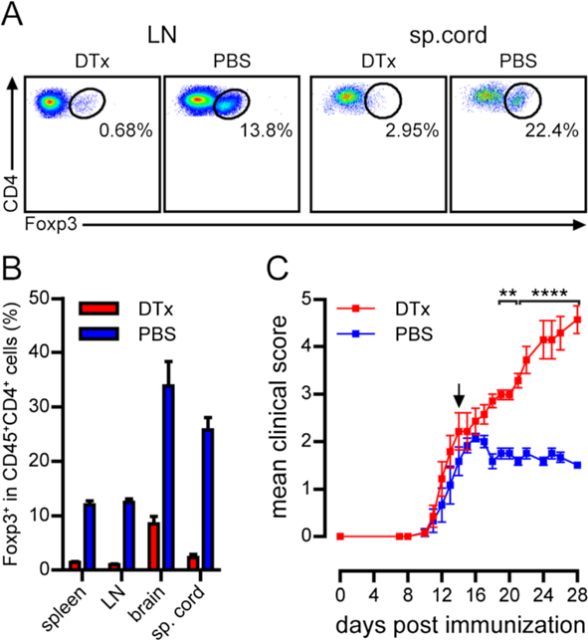
**Exacerbation of EAE after acute T**_**reg**_ **depletion at the peak of the disease.** Representative flow cytometry plots **(A)** or mean frequency (± SEM) of Foxp3^+^ in CD45^+^CD4^+^ T cells **(B)** isolated from indicated organs of DEREG mice two days after DTx or PBS treatment are shown (*n* = 11 mice per group, pooled data from four independent experiments). **(C)** Mean clinical score (± SEM) of mice following immunization and DTx or PBS treatment (day 4 post EAE onset). (*n* = 6-7 mice per group, representative data from two independent experiments. **P < 0.01, ****P < 0.0001, 2way ANOVA).

Our approach to analyze the role of T_reg_*in vivo* during EAE differs from previous attempts which have used anti-CD25 antibodies [9,14-16]. Since these antibodies persist in the circulation for long time, their effect on CD25-expressing activated T_eff_ cannot be excluded. To investigate the functional role of T_reg_ in the CNS during peak EAE, we chose DEREG mice which express a diphtheria toxin (DTx) receptor-enhanced GFP fusion protein, under the control of the *Foxp3* gene locus, permitting specifically timed depletion of T_reg_ by treatment with DTx [6]. We immunized DEREG mice with MOG_35–55_ in CFA and treated them with DTx or PBS during peak of the disease. We monitored the efficiency of T_reg_ depletion in peripheral immune organs and the CNS by flow cytometry. Staining for Foxp3 revealed that T_reg_ population was almost completely absent in DTx-treated DEREG mice compared to control animals in all the organs tested (Figure 1A,B). Next, immunized and treated mice were monitored daily for clinical score to assess the effect of T_reg_ depletion on EAE pathogenesis. While PBS-treated DEREG mice partially recovered from EAE, DTx-treated DEREG mice not only failed to recover from the disease, but also developed severe and, eventually, fatal EAE (Figure 1C). To rule out any adverse effect of DTx on EAE disease course, we have treated MOG_35–55_ immunized wild type mice with DTx during peak EAE. Unlike in DEREG mice, the EAE course was not affected compared to control mice (Additional file 1: Figure S1). These findings suggest that T_reg_ contribute to recovery from EAE and perhaps have an essential function within the CNS.

**Figure 2:**
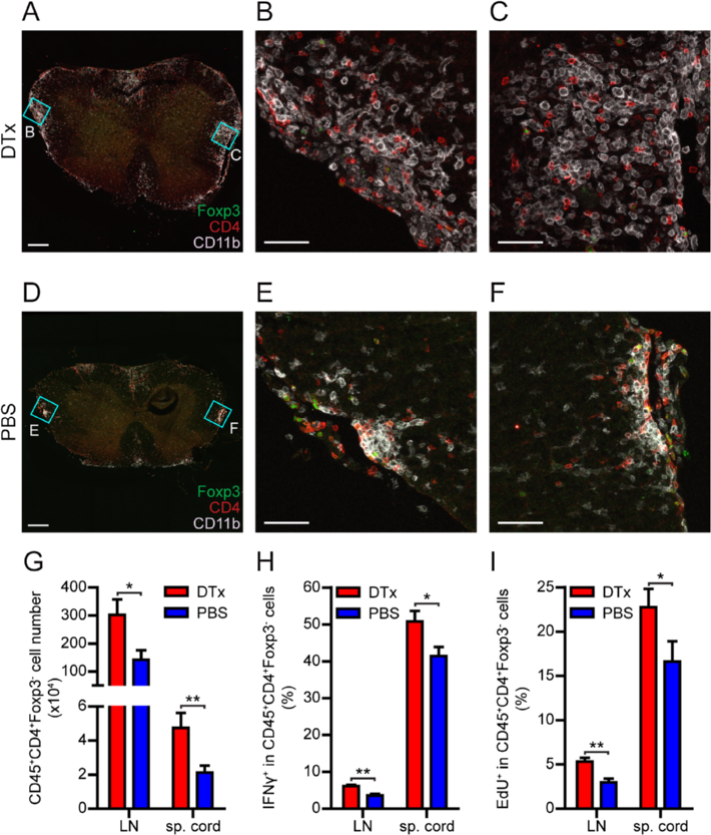
**Increased numbers and enhanced proliferation of T**_**eff**_ **in T**_**reg**_ **-depleted mice. (A)**, **(D)** Representative panoramic pictures of spinal cord cryosections from mice treated with DTx **(A)** or PBS **(D)** stained with anti-Foxp3 (green), anti-CD4 (red) and anti-CD11b Ab (grey) (scale bar: 200 μm). **(B)**, **(C)**, **(E)**, **(F)** Magnified pictures of the indicated regions **(A)** and **(D)** (scale bar: 50 μm). **(G)** Mean absolute numbers (± SEM) of T_eff_ (CD45^+^CD4^+^Foxp3^−^ cells) isolated from LN and spinal cord of DEREG mice two days after DTx or PBS treatment (*n* = 11 mice per group, pooled data from three independent experiments). **(H)** Mean frequency (± SEM) of IFNγ^+^ cells in T_eff_ isolated from LN and spinal cord of DEREG mice two days after DTx or PBS treatment (*n* = 5 mice per group, representative data from three independent experiments) **(I)** EdU was injected i.p. one day after DTx or PBS treatment and 16 hours later lymphocytes were isolated and stained for EdU. Mean frequency (± SEM) of EdU^+^ cells within T_eff_ (*n* = 13-14 mice per group, pooled data from four independent experiments). (*P < 0.05, **P < 0.01, *t*-test).

To learn how T_reg_ ablation led to dramatic disease exacerbation, we stained spinal cord sections of the mice two days after the treatment with DTx or PBS. We observed highly increased numbers of T_eff_, as well as macrophages, in the spinal cord infiltrates in T_reg_-depleted mice compared to the control animals (Figure 2A-F). Next, we quantified the numbers of CD45^+^CD4^+^Foxp3^−^ T cells (T_eff_) in the draining lymph nodes (LN), as well as in the spinal cord, using flow cytometry. Interestingly, lymph nodes and spinal cord of T_reg_-depleted mice contained significantly elevated numbers of T_eff_ (defined as CD45^+^CD4^+^Foxp3^−^ T cells) compared to control mice (Figure 2G). In addition, DTx-treated mice displayed higher frequencies of pro-inflammatory cytokine IFNγ-secreting T_eff_ in the CNS and LN (Figure 2H). In contrast, we detected similar frequencies of IL-17^+^ T_eff_ in both groups. We also did not observe differences in the percentage of activated CD25^+^CD44^high^ T_eff_ in DTx-treated mice (Additional file 1: Figure S2). Therefore, the exacerbated EAE pathology observed in T_reg_-depleted mice could be attributed to increased fraction of IFNγ-producing T_eff_ in the CNS.

T_reg_ are known to suppress the proliferation and activation of T_eff_ cells through multiple mechanisms [1]. Lack of functional T_reg_ results in a lymphoproliferative disease, as in *scurfy* mutant mice [30]. Similar fatal lymphoproliferative disease was observed after chronic depletion of T_reg_ in adult and neonatal mice [5,6]. To determine if the elevated numbers of T_eff_ were a result of increased T cell proliferation, we assessed the *in vivo* proliferation of T_eff_ in the presence or absence of T_reg_. One day after the DTx or PBS treatment of immunized DEREG mice, we injected EdU (5-ethynyl-2´-deoxyuridine), a thymidine analogue which is readily incorporated into cellular DNA during DNA replication, and examined the EdU incorporation in T cells by flow cytometry (Figure 2I). The fraction of EdU^+^ T_eff_ was significantly higher in both LN and the spinal cord of DTx-treated mice compared to control mice, suggesting that the T_eff_ proliferation during EAE is enhanced in the absence of T_reg_.

Since we observed an increased proliferation of T_eff_ in the CNS, we focused on the role of IL-2, a pivotal cytokine for T cell proliferation. T_reg_ express high levels of high affinity IL-2 receptor α (CD25), thereby restricting the availability of IL-2 by direct consumption to restrain the activation of proliferating T cells [31]. We hypothesized that the enhanced T_eff_ proliferation that we observed after elimination of T_reg_ could be attributed to increased availability of IL-2. To test this hypothesis, we quantified the IL-2 protein levels in LN and spinal cord tissue extracts from T_reg_-depleted and T_reg_-intact mice with EAE. However, both groups exhibited similar levels of IL-2 (Additional file 1: Figure S3A). Furthermore, administration of anti-IL-2 blocking antibodies in parallel with DTx treatment did not prevent EAE exacerbation (Additional file 1: Figure S3B). These findings suggest that IL-2 deprivation is not a major mechanism used by T_reg_ to control T_eff_ proliferation *in vivo* within the CNS during EAE.

We considered the possibility of direct or indirect interactions of T_reg_ with T_eff_ and APCs to mediate suppression of T_eff_ in the CNS during EAE recovery. Previous 2-photon imaging studies in LN have shown that T_reg_ can limit the contacts between T_eff_ and DCs [21-23]. However, the effect of T_reg_ on the migratory behavior of T_eff_ within the CNS during EAE is not known. We sought to investigate how the ablation of T_reg_ can affect the dynamic behavior of T_eff_ in the CNS using intravital two-photon imaging. To this end, we crossed T-Red mice, in which T cells express the red fluorescent protein dsRedII [32], to DEREG mice. Subsequently, we treated MOG-immunized T-Red x DEREG mice with DTx or PBS at the peak of EAE and performed intravital two-photon imaging in the spinal cord meninges.

**Figure 3:**
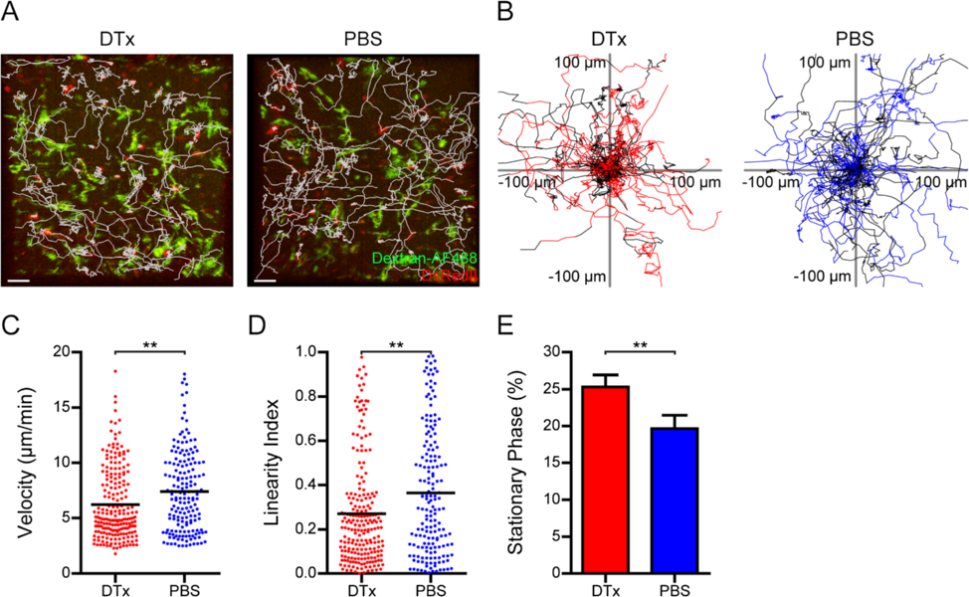
**T**_**eff**_ **dynamics in the CNS of T**_**reg**_ **-depleted mice.** Intravital two-photon imaging was performed in the spinal cord meninges of T-Red x DEREG mice two days after DTx or PBS treatment. **(A)** Trajectories (white lines) of dsRedII^+^ T_eff_ overlaid with snapshots from representative videos. One representative out of three independent experiments per treatment condition is shown (red: T_eff_, green: APC, scale bar: 30 μm) **(B)** Superimposed trajectories of T_eff_ movements after DTx or PBS treatment. Time points with contacts with APC are indicated in red or blue, respectively. One representative out of three independent experiments per treatment condition is shown. **(C)** Average velocity, **(D)** linearity index (sum of the total displacement divided by the path length of a cell), **(E)** stationary phase (part of the track with velocity <3 μm/min) (± SEM) of T_eff_ after DTx or PBS treatment (pooled data from three independent experiments (**P < 0.01, *t*-test) (pooled data from three independent experiments).

Cell tracking of the dsRedII-expressing T_eff_ revealed that T_eff_ displayed more confined trajectories in the CNS of DTx-treated mice than PBS-treated mice (Figure 3A,B). Indeed, analysis of the T_eff_ tracks showed that the cells had significantly reduced mean velocity and linearity index compared to the control animals in the CNS (Figure 3C,D). In parallel, the stationary phase of T_eff_ was increased (Figure 3E). Collectively, these findings suggest that the absence of T_reg_ results in decreased motility of T_eff_, which indicates enhanced interactions with potential APCs within the inflamed spinal cord meninges.

In summary, using 2-photon imaging, we showed that T_reg_ exert dynamic control over T_eff_ within the CNS during effector phase of EAE. This finding doesn’t exclude additional actions mediated by T_reg_ in the periphery. Our results are in agreement with many reports which showed that ablation of T_reg_ population (by treatment with anti-CD25 antibody) exacerbates EAE [9,14-16]. However, a major disadvantage of this approach is that CD25 is not a T_reg_-specific marker, but is also expressed by activated T_eff_ complicating the interpretation of these findings. Our approach using DEREG mice circumvents these issues by specifically timed depletion of T_reg_. This is also a first study in an active EAE which uses specific T_reg_ depletion. Our results are compatible with a recent report which showed that selective Treg depletion resulted in an increased incidence and accelerated disease onset in a spontaneous EAE model [19].

While the importance of Treg during CNS autoimmunity is unequivocally shown, where and in which phase of the disease they are important is not clear. The main conclusion from our study is that the T_reg_ exert their regulatory control over T_eff_ within the CNS in addition to their known peripheral effects. At first glance, our results are in contrast to a report by Korn *et al.*, which suggested that regulatory T cells accumulate in the CNS but are unable to control CNS infiltrating T_eff_ during peak of the disease [8]. The conclusions were drawn based on the inability of CNS derived T_reg_ to suppress T_eff_ proliferation. We, however, have followed the behavior of T_eff_ cells in their “native” environment. Moreover, several studies reported that natural recovery from EAE correlating with increasing T_reg_ numbers suggests that T_reg_ are essential to mediate recovery [8-10]. Concerning the potential mode of action, we observed that the exacerbation of EAE was preceded by an increase in the numbers of T_eff_ due to local proliferation in the absence of T_reg_. Earlier reports using two-photon microscopy have demonstrated that CD4^+^CD25^−^ T cells established longer contacts with DCs in lymph node in the absence of T_reg_ (defined as CD4^+^CD25^+^ T cells), while in T_reg_-sufficient environment these contacts were inhibited [22,23]. Moreover, T_reg_ have been recently described to suppress the T cell movements in the LN during EAE in a PSGL-1-dependent mechanism [21]. Our results show that there is an increase in the motility of T_eff_ in T_reg_-depleted mice and there was an increase in the stationary phase of T_eff,_ indicating increased contacts with APCs in the CNS. In conclusion, our findings suggest that T_reg_ are indispensable for recovery from EAE through their actions within and outside of the CNS.

## Additional file

Additional file 1: Figure S1. DTx injection does not affect EAE course in non-transgenic mice. Mean clinical score (± SEM) of mice following immunization and DTx or PBS treatment (day 4 post EAE onset). (*n* = 5-6 mice per group, representative data from two independent experiments. **Figure S2.** Expression of IL-17 and T cell activation markers in T_reg_-depleted mice. (A) Mean frequency (± SEM) of IL-17^+^ cells in T_eff_ isolated from LN and spinal cord of DEREG mice two days after DTx or PBS treatment (*n* = 5 mice per group, representative data from three independent experiments) (B) Mean frequency (± SEM) of CD25^+^CD44^high^ cells within T_eff_ isolated from LN and spinal cord of DEREG mice two days after DTx or PBS treatment (*n* = 8 mice per group, pooled data from two independent experiments). **Figure S3.** T_reg_ do not use IL-2 deprivation to limit T_eff_ proliferation. **(A)** IL-2 protein levels (± SEM) in LN and spinal cord of DEREG mice two days after DTx or PBS treatment (*n* = 5 mice per group, pooled data from two independent experiments). **(B)** Mean clinical score (± SEM) of mice following immunization and DTx treatment (day 4 post EAE onset) as well as anti-IL2 antibody treatment on days 4 and 6 post EAE onset (see arrow) (*n* = 3-4 mice per group, representative data from two independent experiments).
