# Supplementary figures and images for: Treg cells mediate recovery from EAE by controlling effector T cell proliferation and motility in the CNS
Source: Acta Neuropathol Commun. 2014 Dec 5;2:163. doi: 10.1186/s40478-014-0163-1 (PMC4268825; doi:10.1186/s40478-014-0163-1)

Supplementary Fig. 1

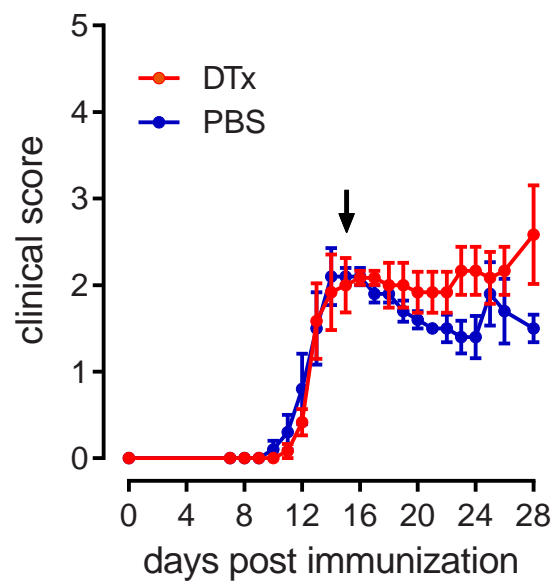

Supplementary Fig. 2

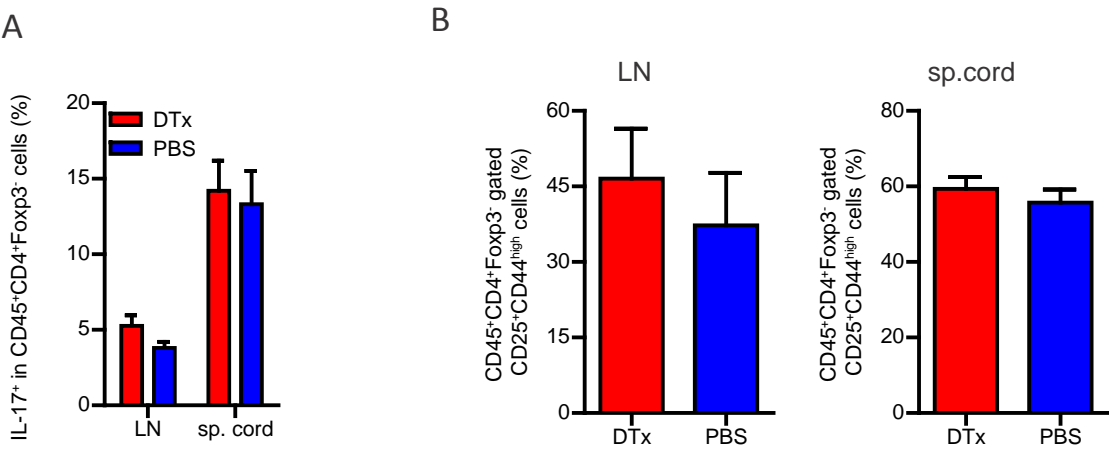

Supplementary Fig. 3

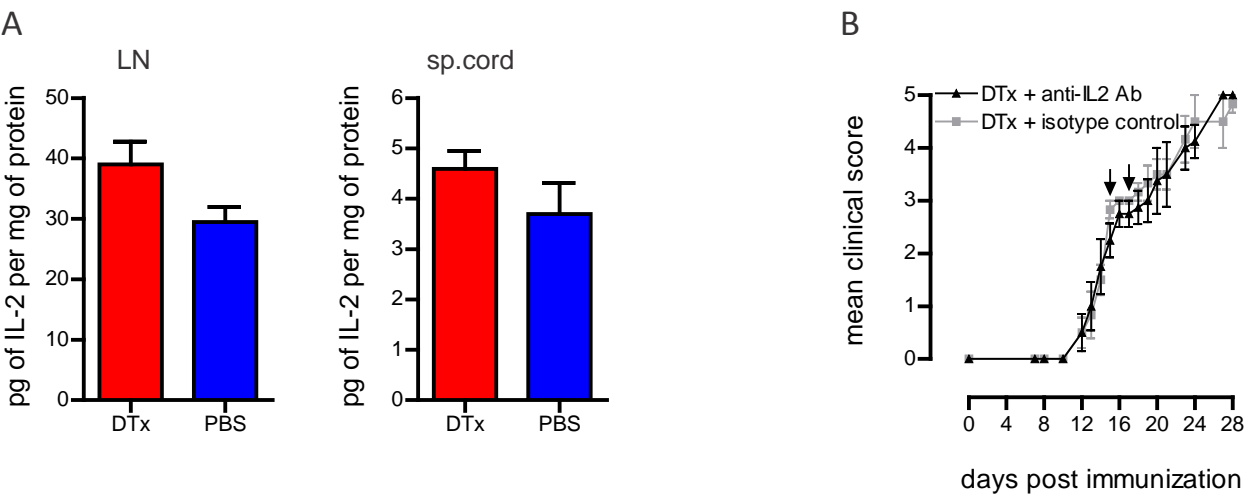

Supplement: Additional file 1: Figure S1. — DTx injection does not affect EAE course in non-transgenic mice. Mean clinical score (± SEM) of mice following immunization and DTx or PBS treatment (day 4 post EAE onset). (n = 5-6 mice per group, representative data from two independent experiments. Figure S2. Expression of IL-17 and T cell activation markers in Treg-depleted mice. (A) Mean frequency (± SEM) of IL-17+ cells in Teff isolated from LN and spinal cord of DEREG mice two days after DTx or PBS treatment (n = 5 mice per group, representative data from three independent experiments) (B) Mean frequency (± SEM) of CD25+CD44high cells within Teff isolated from LN and spinal cord of DEREG mice two days after DTx or PBS treatment (n = 8 mice per group, pooled data from two independent experiments). Figure S3. Treg do not use IL-2 deprivation to limit Teff proliferation. (A) IL-2 protein levels (± SEM) in LN and spinal cord of DEREG mice two days after DTx or PBS treatment (n = 5 mice per group, pooled data from two independent experiments). (B) Mean clinical score (± SEM) of mice following immunization and DTx treatment (day 4 post EAE onset) as well as anti-IL2 antibody treatment on days 4 and 6 post EAE onset (see arrow) (n = 3-4 mice per group, representative data from two independent experiments). [file 40478_2014_163_MOESM1_ESM.pdf]
